# Biocompatibility of a Zr-Based Metallic Glass Enabled by Additive Manufacturing

**DOI:** 10.1021/acsabm.2c00764

**Published:** 2022-12-02

**Authors:** Lisa Larsson, Jithin James Marattukalam, Eirini-Maria Paschalidou, Björgvin Hjörvarsson, Natalia Ferraz, Cecilia Persson

**Affiliations:** † Department of Materials Science and Engineering, Biomedical Engineering, Box 534, Uppsala University, SE- 75121Uppsala, Sweden; ‡ Department of Physics, Materials Physics, Box 530, Uppsala University, SE-75121Uppsala, Sweden; § Department of Chemistry Ångström, Box 538, Uppsala University, SE-751 21Uppsala, Sweden; ∥ Department of Materials Science and Engineering, Nanotechnology and Functional Materials, Box 35, Uppsala University, SE- 75103Uppsala, Sweden

**Keywords:** AMLOY-ZR01, bulk metallic glass, additive manufacturing, selective laser melting, MC3T3, surface roughness, ion release profile

## Abstract

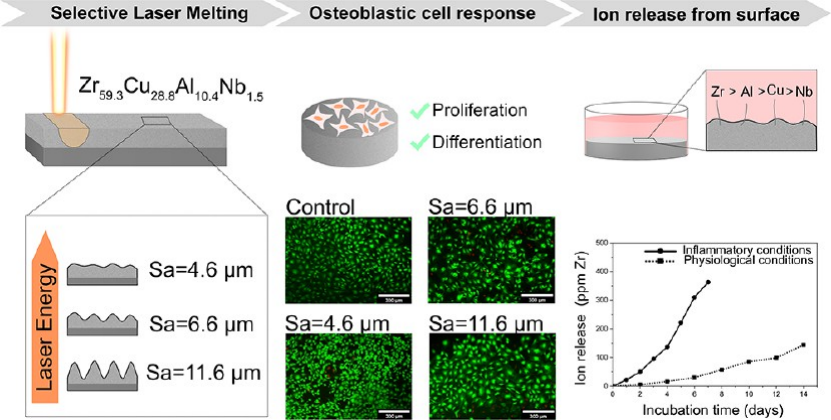

The present work explored the use of the selective laser melting (SLM) technique to develop a Zr-based bulk metallic glass (BMG) and investigate the influence of the process parameters on obtaining different levels of surface roughness. Moreover, the potential of the additively manufactured BMG Zr_59.3_Cu_28.8_Al_10.4_Nb_1.5_ (trade name AMLOY-ZR01) as an implant material was studied by evaluating the osteoblastic cell response to the alloy and its stability under simulated biological environments. The materials were characterized in terms of degree of crystallinity, surface roughness, and morphology, followed by a systematic investigation of the response of the MC3T3-E1 preosteoblastic cell line to the as-printed samples. The materials supported cell proliferation and differentiation of the preosteoblastic cells, with results comparable to the reference material Ti-6Al-4V. The surface microroughness and surface morphology (porous or groove-type laser tracks) investigated in this study did not have a significant effect on modulating the cell response. Ion release experiments showed a large increase in ion release under inflammatory conditions as compared to regular physiological conditions, which could be attributed to the increased local corrosion under inflammatory conditions. The findings in this work showed that the surface roughness of the additively manufactured BMG AMLOY-ZR01 can be tailored by controlling the laser power applied during the SLM process. The favorable cell response to the as-printed AMLOY-ZR01 represents of a significant advancement of the investigation of additively manufactured BMGs for orthopedic applications, while the results of the ion release study highlights the effect that inflammatory conditions could have on the degradation of the alloy.

## 1 Introduction

Metallic materials play a significant role in the biomedical field, particularly as orthopedic implants owing to their mechanical properties. The most commonly used metallic biomaterials for implants include stainless steel, titanium and its alloys, and cobalt-chromium alloys.^1−4^ However, conventionally used orthopedic implants suffer from certain shortcomings, of which a high elastic modulus, e.g., can be a major setback for load-bearing applications, as it gives rise to stress shielding, which may cause bone resorption and complications at the tissue/implant interface.^5,6^ Therefore, having a Young’s modulus of the implant as close as possible to that of bone would be advantageous for the long-term clinical performance of the implant.

Bulk metallic glasses (BMGs) are a relatively new class of metallic materials developed over the past couple of decades with the first BMG being developed during the 80s.^7^ Unlike conventional crystalline materials, BMGs exhibit no long-range atomic order and have shown a promising potential for biomedical applications.^8−10^ A combination of high strength, excellent corrosion and wear resistance, and relatively low Young’s modulus (80–100 GPa),^11^ arising from the unique disordering of the atomic structure,^12^ make BMGs potential candidates for orthopedic applications and particularly for load-bearing implants.^13^ Despite advancements in the development of biomedical BMGs made during the past decade, the production of BMGs in larger dimensions and complex geometries has remained challenging. Conventional processing routes such as suction casting, squeeze casting, melt spinning, etc., impose size restrictions as the solidification rates needed to form the amorphous structure is very high. Indeed, the typical critical casting thickness of BMGs is of the order of a few millimeters.^14^ However, additive manufacturing (AM) through selective laser melting (SLM) can be used to circumvent the size limitations and allow fabrication of components much larger than the critical casting thickness of the material.^15^ The cooling rates during an SLM process can be as high as 10^4^–10^7^ K/s,^16^ far exceeding the critical cooling rates required for formation of the amorphous phase in most BMG systems, thus assuring the amorphous structure in the final printed components.

Furthermore, SLM offers flexibility in the manufacturing of biomedical implants as customized parts can be produced in a relatively straightforward manner: complex shapes resembling the damaged part can be produced using computed tomography images, allowing for patient-specific implants.^17^ Complex internal structures with gradient porosities, mimicking the natural bone structure, can also be produced by AM.^18−20^ This approach ensures the right distribution of compressive stiffness across various regions, which are virtually impossible to prepare by any traditional manufacturing method.

Over the recent years, BMGs based on zirconium have in particular been highlighted as viable candidates for biomedical implants^21−23^ and alloy compositions free of nickel are of specific interest^8,24^ because of the severe side effects that may arise from nickel allergy following implantation of nickel-containing biomaterials.^25^ A series of nickel-free Zr-based BMGs with good glass forming ability were developed by Jin and Löffler,^26^ demonstrating the absence of cytotoxicity in Ni-free BMG Zr_58_Cu_22_Fe_8_Al_12._^27^ More recently, a Zr-based BMG with composition Zr_60.14_Cu_22.31_Fe_4.85_Al_9.7_Ag_3_, developed by Liu et al.,^28^ showed an exceptional glass forming ability, low Young’s modulus, and high corrosion resistance, together with good bone cell adhesion behavior.^29^ Similar observations were also made by Li et al. in a systematic study on the biocompatibility of Ni-free Zr_61_Ti_2_Cu_25_Al_12_ BMG, reporting that the proliferation of several relevant cell types (fibroblast, endothelial and osteoblast cells) on the metallic glassy surface was as good as that of pure Ti and Ti64 alloy.^30^

A Ni-free Zr-based BMG with the composition Zr_59.3_Cu_28.8_Al_10.4_Nb_1.5_ (trade name AMLOY-ZR01) was selected in this study to explore its potential application as a biomedical implant. Previous studies on additively manufactured Zr_59.3_Cu_28.8_Al_10.4_Nb_1.5_ have demonstrated useful mechanical properties of the material for structural applications.^31,32^ In addition, Bordeenithikasem et al. reported the potential application of AMLOY-ZR01 as a wear-resistant coating in gears and bearings.^33^ Despite the promising mechanical, wear, and corrosion properties, a systematic investigation of the biocompatibility has never been conducted for this alloy. Moreover, investigations about the alloy corrosion behavior and ion release in biological environments are lacking. If used as an implant material, AMLOY-ZR01 will be exposed to complex biological environments, which may affect the wear and corrosion properties. Surrounding factors such as the presence of chloride and other ions, pH, and biomolecules could affect the corrosion and metal ion release of the BMG alloy.^34^ Furthermore, the inflammatory reaction taking place during biomaterial implantation, characterized by a decrease in the surrounding pH and high levels of reactive oxygen species (ROS), represents a challenge for the stability of metal and metal alloys.^35,36^

A strategy for directing cell-material interactions, thus modulating the material biocompatibility, is to control the material surface roughness. However, to predict the specific effect of the material surface roughness on cell response remains a challenge.^37^ In the case of metal/alloy biomaterials and bone cells, rougher surfaces have been related with higher cell proliferation and differentiation^38−40^ in some cases, while other studies report the opposite effect or no significant effect of surface roughness on the response of bone cells.^41,42^ Moreover, studies regarding BMG surface roughness and cell response are scarce.^43−45^

In the present work, we demonstrate the use of SLM to fabricate the Ni-free AMLOY-ZR01 BMG and investigate the material’s biocompatibility and ion release, bearing in mind its potential application as bone implant. The laser power applied during the SLM process was tuned to control the surface roughness of the additively manufactured AMLOY-ZR01 BMG. The preosteoblastic cell line MC3T3-E1 was selected to investigate the cell interactions with three sets of as-printed AMLOY-ZR01 samples presenting different surface roughnesses and morphologies. The *in vitro* cell response to the materials was evaluated by performing indirect cytotoxicity tests followed by *in vitro* cell proliferation and cell differentiation studies. In addition, ion release experiments were performed in simulated regular physiological conditions and simulated inflammatory conditions to investigate the stability of the BMG in the biological environment.

## 2 Materials and Methods

### 2.1 Sample Fabrication

#### 2.1.1 Selective Laser Melting

Zr_59.3_Cu_28.8_Al_10.4_Nb_1.5_ (trade name AMLOY-ZR01) gas-atomized powders were produced and provided by Heraeus, GmbH, using industrial-grade feedstock materials. The D50 and D90 of the powder particles were 25 μm and 44 μm respectively. The as-printed samples were manufactured using an EOS M100 (EOS GmbH, Germany) system equipped with a continuous ytterbium fiber laser. The process parameters were selected based on a previous study.^32^ Samples were fabricated with a step size of 20 W laser power at 55, 75, and 95 W within an optimal processing window to obtain samples with different morphologies and surface roughnesses. The as-printed samples are hereinafter referred to by the respective laser power used in their fabrication. The layer thickness was set to 20 μm for each powder bed deposition with a hatch spacing of 100 μm and scan speed of 2000 mm/s. Argon was used as the process gas during the SLM process to minimize the oxygen contamination in the samples. The samples were printed using a remelting scan strategy (each layer melted twice) with 67° rotations between each layer.

#### 2.1.2 Suction Casting

The as-cast samples used in the present study were fabricated by suction casting by Heraeus additive Manufacturing GmBH. High purity elements (>99.9%) were used as raw materials, and the master alloy was synthesized using electric arc melting. The suction casting was performed by remelting the ingot of the master alloy and casting it into a water-cooled copper mold, with a rod shape of 5 mm diameter. The entire process of electric arc melting and suction casting was performed under a high purity Ti getter and an argon atmosphere.

### 2.2 Sample Characterization

#### 2.2.1 Pycnometry

The density of the as-printed samples was measured with respect to a conventionally as-cast sample of AMLOY-ZR01. The weight of the sample (*W*_1_) was measured in air at room temperature (RT). The samples were then immersed in water, and the weight (*W*_2_) was measured at RT. The relative density of the sample was calculated by Archimedes’ principle as follows:
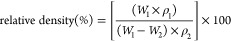


The density of water at RT (ρ_1_) was taken to be 0.9980 g/cm^3^. The absolute density (ρ_2_) of conventionally cast AMLOY-ZR01 (6.64 g/cm^3^), measured using Archimedes’ principle, was used as the reference. The weight of the samples was measured using a high-precision (0.1 mg) electronic balance (Mettler Toledo AB204-S).

#### 2.2.2 X-ray Diffraction

The printed samples were investigated by X-ray diffraction to study the influence of laser power on the phase formation during SLM. The X-ray measurements were performed with Cu Kα radiation, using a Bruker D8 advanced diffractometer operating at 40 kV and 40 mA. The 2θ angle was varied from 30 to 90°. A detailed structural and compositional study performed on as-printed AMLOY-ZR01 samples can be found elsewhere.^32^

#### 2.2.3 Optical Interferometry

The surface roughness of the three different sets of as-printed samples were measured with a Zygo Nexview NX2 3D optical profilometer, equipped with a 5-axis motorized stage (Zygo corporation Middlefield, CT), based on the principle of white light interference.^46^ The data was acquired using Mx software under the following operating conditions: 10× Michelson objective lens with a 0.5× zoom and a scan length of 100 μm. The roughness parameters *S*_a_ and *S*_z_ were generated by Mx from the interference data. For each sample set, a total of 10 measurements were made and the roughness parameters (*S*_a_ and *S*_z_) were reported with their corresponding average and standard deviation.

#### 2.2.4 Scanning Electron Microscopy

Scanning electron microscopy (SEM) was used to characterize the surface morphology and porosity of the three different sets of as-printed samples. The samples were cleaned using ethanol (99%) in an ultrasonic bath for 10 min, dried, and mounted on conductive carbon tapes before loading them into the SEM chamber for evaluating the surface morphology. Scanning electron micrographs were taken at an accelerating voltage of 5 kV with a Zeiss 1550 SEM, equipped with a secondary electron (SE2) detector at a working distance of 10 mm.

### 2.3 *In Vitro* Cell Studies

The murine preosteoblastic cell line MC3T3-E1, subclone 14 (CRL-2594, American Type Culture Collection (ATCC)), a well-known cell model to test biomaterials in bone tissue engineering related research,^47^ was selected for the *in vitro* cell studies. Cells were maintained in alpha minimum essential medium (α-MEM, Gibco, USA) supplemented with 10% fetal bovine serum (FBS) (Gibco, USA), 100 IU/mL penicillin, and 100 μg/mL streptomycin (Gibco, USA), hereinafter referred to as proliferation medium. Cell cultures were kept in a 37 °C and 5% CO_2_ incubator with a humidified atmosphere and subcultured at 80% confluency.

#### 2.3.1 Indirect Cytotoxicity Test

An indirect cytotoxicity test was carried out to evaluate potential toxic effects of the AMLOY-ZR01 materials due to leaching. The test was performed following the ISO standard 10993-5.^48^ As-printed AMLOY-ZR01 discs (13 mm diameter and 5 mm height) were immersed in 0.55 mL of proliferation medium per disc and incubated at 37 °C, 5% CO_2_ for 24 h. Simultaneously, MC3T3-E1 cells were seeded in 96-well culture plates (VWR, USA) at a cell density of 2.5 × 10^4^ cells/cm^2^ and cultured for 24 ± 2 h in a 37 °C, 5% CO_2_ incubator with a humidified atmosphere. Thereafter, the near confluent cell monolayers were exposed to the extracts (undiluted, 1:2 and 1:5 diluted in fresh cell culture medium) for 24 ± 2 h in a 37 °C, 5% CO_2_ incubator with a humidified atmosphere. Cells cultured in cell culture medium served as the negative control and cells exposed to 5% dimethyl sulfoxide (DMSO) in cell culture medium served as the positive control. After exposure, cell viability was evaluated by the alamarBlue (AB) assay. Cell culture medium was removed, the cell layers were carefully washed with phosphate buffered saline (PBS, Gibco, USA), and 200 μL of the AB reagent (Thermo Fisher Scientific, USA) diluted 1:10 in cell culture medium was added per well. After 90 min of incubation in a 37 °C and 5% CO_2_ incubator with a humidified atmosphere, 100 μL of aliquots from each well were transferred to a black 96-well plate and the fluorescence intensity was measured by a spectrofluorometer (Tecan Infinite 200 plate reader, Tecan, Switzerland) at an excitation wavelength of 560 nm and emission wavelength of 590 nm. Four independent experiments were performed, and samples were run in triplicate. Results were expressed as percentage of cell viability with respect to the negative control.

#### 2.3.2 Cell Proliferation Study

The ability of the as-printed AMLOY-ZR01 materials to promote cell proliferation was evaluated by culturing MC3T3-E1 cells on the surface of the material’s discs up to 7 days. AMLOY-ZR01 discs (5 mm in diameter and 5 mm in height) were placed in 96-well plates and precoated with 200 μL of cell proliferation medium for 6 h. After the precoating, the medium was removed and 200 μL of cell suspension was added to the wells, ensuring a cell density of 10,000 cells/cm^2^, and cultured in a 37 °C and 5% CO_2_ incubator with a humidified atmosphere. The cell culture medium was changed every 2–3 days. The number of adherent cells, cell viability, and cell morphology were evaluated after 1, 3, and 7 days of culture. Titanium grade 5 discs (Ti-6A1-4V, Peter Brehm, Germany) were used as reference material, it already being an established biomaterial. Cells cultured in tissue culture plate wells were used as the proliferation control. Samples of conventionally manufactured as-cast AMLOY-ZR01 were also included as cell culture substrates when evaluating the number of adherent cells and cell morphology.

##### 2.3.2.1 Lactate Dehydrogenase Assay

The number of adherent cells at the different time points was evaluated by measuring the activity of the intracellular enzyme lactate dehydrogenase (LDH) in the cell lysates. The material discs were transferred to a new 96 well plate; cells were carefully washed with PBS and thereafter lysed by adding 200 μL of CelLytic M cell lysis reagent (Sigma-Aldrich, Germany) followed by 15 min incubation under agitation (625 rpm) and one cycle of freeze–thaw. Cell debris was eliminated by centrifuging the lysates at 6100*g* for 15 min. LDH activity was measured by the LDH-Cytotoxicity Assay kit II (Abcam, United Kingdom), following the manufacturer’s guidelines. Briefly, 10 μL of sample was incubated with 100 μL of the LDH kit reaction mix for 30 min at room temperature and protected from light. After incubation, 10 μL of stop solution was added to each well and the absorbance at a wavelength of 450 nm with a reference wavelength of 650 nm was measured using a Tecan Infinite 200 plate reader (Tecan, Switzerland). The experiments were conducted at least four times, with triplicate wells for each sample. Results were expressed as arbitrary units normalized by the surface area of the culture substrates. Control experiments performed in the absence of cells showed that the AMLOY-ZR01 samples did not interfere with the LDH assay.

##### 2.3.2.2 Live/Dead Staining

Cell attachment and cell viability at the different time points were assessed qualitatively by live/dead staining of the attached cells, followed by fluorescence microscopy imaging. A total of 200 μL of staining solution (2 μL calcein-AM and 1 μL of propidium iodide per mL of PBS) was added to each well, and the well plate was incubated for 15 min at 37 °C. After incubation, cells were observed using a fluorescence microscope (Nikon eclipse Ti-U, Nikon, Japan).

##### 2.3.2.3 Scanning Electron Microscopy of Cells

The morphology of adherent cells was evaluated by SEM imaging of the samples after 1, 3, and 7 days of cell proliferation. Material discs with adherent cells were rinsed with PBS and cells fixed with 2.5% (v/v) glutaraldehyde in PBS. Samples were then dehydrated through a series of ethanol concentrations [10, 30, 50, 70, 90, and 100% (v/v)], followed by incubations with hexamethyldisilazane (HMDS) solutions (HMDS 1:2 ethanol, HMDS 2:1 ethanol, and 100% HMDS). After dehydration, samples were air-dried and coated with gold using the SC7640 Sputter Coater (Thermo VG Scientific, USA) for 20 s at 20 mA before SEM imaging. To facilitate the SEM imaging, the tissue culture plate well used as proliferation control was substituted by 13 mm Thermanox (TMX) discs (Thermo Scientific, USA). Cell morphology was evaluated using a Leo 1550 SEM instrument (Zeiss, Germany).

#### 2.3.3 Cell Differentiation Study

To evaluate the effect of the as-printed AMLOY-ZR01 materials on cell differentiation, MC3T3-1 cells were cultured on the material discs under differentiation conditions and the cell response in terms of the activity of the early osteogenic marker alkaline phosphatase (ALP) was evaluated. Discs (5 mm diameter, 5 mm height) were placed in 96-well plates and precoated with 200 μL of proliferation medium for 6 h. After the precoating, the medium was removed and 200 μL of cell suspension in proliferation medium was added to the wells, corresponding to a cell density of 7.5 10^4^ cells/cm^2^. After 3 days of cell proliferation, the medium was changed to a differentiation medium, which consisted of minimum essential medium alpha modification (MEM Alpha Modification, HyClone, USA), supplemented with 10% FBS, 100 IU/mL penicillin, and 100 μg/mL streptomycin and the differentiation factors ascorbic acid (50 μg/mL) and glycerophosphate (10 mM). The differentiation medium was changed every 2–3 days. ALP activity was measured after 3, 7, and 10 days of culture in the differentiation medium. Titanium grade 5 discs (Ti-6A1-4V, Peter Brehm, Germany) were used to compare the cell differentiation response of cells on the AMLOY-ZR01 material to the already well-established titanium biomaterial. Cells cultured in tissue culture plate wells were used as the differentiation control.

##### 2.3.3.1 ALP Activity Assay

ALP activity was measured by a colorimetric method based on the conversion of *p*-nitrophenyl phosphate into *p*-nitrophenol by ALP. The material discs were transferred to a new 96 well plate, cells were carefully washed with PBS, and 200 μL of CelLytic M cell lysis reagent (Sigma-Aldrich, Germany) was added, followed by 15 min incubation under agitation (625 rpm) and one cycle of freeze–thaw. Cell debris was eliminated by centrifuging the lysates at 6100*g* for 15 min. For the ALP assay, 50 μL of the cell lysate was placed in a 96-well plate together with 100 μL of the substrate *p*-nitrophenyl phosphate (alkaline phosphatase yellow liquid substrate system, Sigma-Aldrich, Germany). The plate was protected from light and incubated at room temperature for 10–30 min; the final time of incubation was noted for each assay. To stop the reaction, 50 μL of 3 M KOH was added to each well and the production of *p*-nitrophenol was determined by measuring the absorbance at 405 nm and using a standard curve of known concentrations of *p*-nitrophenol. ALP activity was expressed as *p*-nitrophenol concentration normalized by total protein (μg protein/substrate area) and reaction time (minutes).

Total protein in cell lysates was quantified using a micro BCA protein assay kit (Thermo Fisher Scientific, USA) following the manufacturer’s protocol and using a bovine serum albumin standard curve. Briefly, 100 μL of lysate was mixed with 100 μL of the working reagent from the assay kit, incubated at 37 °C for 2 h and the absorbance was measured at 562 nm (Tecan Infinite 200 plate reader, Tecan, Switzerland). A minimum of three independent experiments were performed and samples were run in triplicate.

### 2.4 Ion Release Study

The ion release profile of the AMLOY-ZR01 materials was investigated *in vitro* under two simulated conditions, regular physiological conditions and inflammatory conditions, as described below. Three independent experiments were performed for each condition of surface roughness. At the end of the ion release study, the AMLOY-ZR01 discs were characterized in terms of surface roughness and surface morphology as described in Sections 2.2.3 and 2.2.4, respectively.

#### 2.4.1 Simulated Regular Physiological Conditions

AMLOY-ZR01 discs (34 mm in diameter, 5 mm height) were placed in 6-well plates (Thermo Scientific, USA) and cell culture medium (MEM Alpha Modification, HyClone, USA) supplemented with 10% FBS and 100 IU/mL penicillin and 100 μg/mL streptomycin was added to the wells with an extraction ratio of 6 cm^2^/mL. The covered discs were incubated at 37 °C, 5% CO_2_, and a humidified atmosphere for a total of 14 days. Every 48 h, the medium was collected, and fresh medium was added. The concentration of Zr, Cu, Al and Nb ions in the collected media was analyzed by inductively coupled optical emission spectrometry (ICP-OES) using an Avio 200 instrument (PerkinElmer, USA). The collected samples were diluted with nitric acid to a final acid concentration of 2% (using 65% nitric acid, Emsure ISO, Merck, Germany and ASTM type I water, SPEX Certiprep, Germany) and filtered through 0.45 μm filters (Puradisc 25 AS, Whatman, United Kingdom).

#### 2.4.2 Simulated Inflammatory Conditions

To mimic the situation of acute inflammation that takes place when a material is implanted in the human body, a simulated inflammatory medium was prepared by adding hydrogen peroxide (final concentration 150 mM, Sigma-Aldrich, Germany) to the physiological condition medium described in Section 2.4.1 and the pH was adjusted to 5.2 with 1 M HCl.^36^ The medium was prepared fresh and changed every 24 h for 7 days to keep the reactivity of the medium. The extraction and sample preparation procedure was otherwise identical to the one described above. The samples were analyzed by ICP-OES using the Avio 200 instrument as described above.

### 2.5 Statistical Analysis

All statistical analyses were performed using IBM SPSS statistics software (IBM Corp. Released 2015. IBM SPSS Statistics for Windows, Version 23.0. Armonk, NY: IBM Corp., USA). One-way ANOVA was used to assess statistical differences between groups. Statistical significance was noted at *p* < 0.05. Homogeneity of variance was assessed by Levene’s test, and Tamhane’s post-hoc test was subsequently applied in the case of significance, while Scheffe’s post-hoc test was applied in the case of nonsignificance.

## 3 Results and Discussion

### 3.1 Material Characterization

The as-printed AMLOY-ZR01 samples were characterized in terms of structure, surface morphology, and surface roughness in comparison with the properties of the conventionally manufactured as-cast alloy. The relative densities of the as-printed X-ray amorphous samples were calculated using the Archimedes method in comparison with the as-cast sample and were found to be 99.3, 99.8, and 99.9% for the 55, 75, and 95 W samples, respectively, thus showing that the laser power only marginally influenced the final part density in as-printed samples.

**Figure 1:**
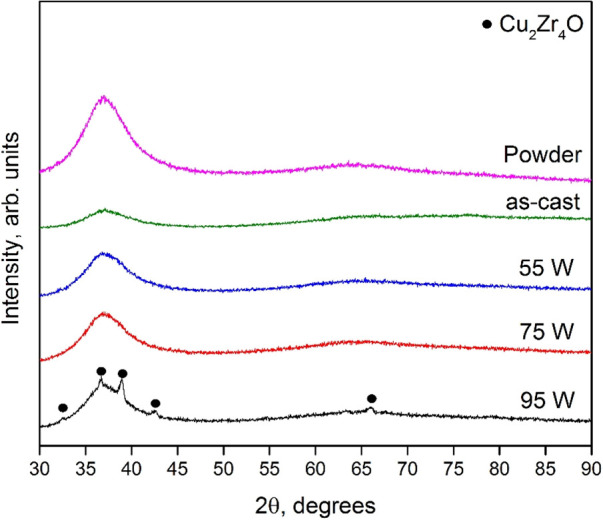
X-ray diffractogram of powder, as-cast and as-printed (55, 75, and 95 W) AMLOY-ZR01 samples. The XRD amorphous samples are characterized by a broad halo around 2θ = 37°. Crystalline reflections originating from metastable cF96 phase (Cu_2_OZr_4_) are observed at a laser power of 95 W. The data are shifted to avoid overlap in the figure.

The influence of the processing on the ordering and phase formation was analyzed using X-ray diffraction. Typical results are shown in Figure 1. The samples printed with laser powers of 55 and 75 W and the as-cast samples exhibit similar results, with a broad scattering maximum at around 2θ = 37° attributed to the amorphous state of the material. However, in the sample processed using 95 W, peaks corresponding to crystalline phases are observed. These reflections arise from scattering from crystalline grains formed in a devitrification of the amorphous phase at higher laser energy intensities during the SLM process. The peaks were identified as arising from Cu_2_Zr_4_O. The results on phase composition described in the present study are consistent with our previous investigation on laser-processed AMLOY-ZR01, as described in ref (32).

**Figure 2:**
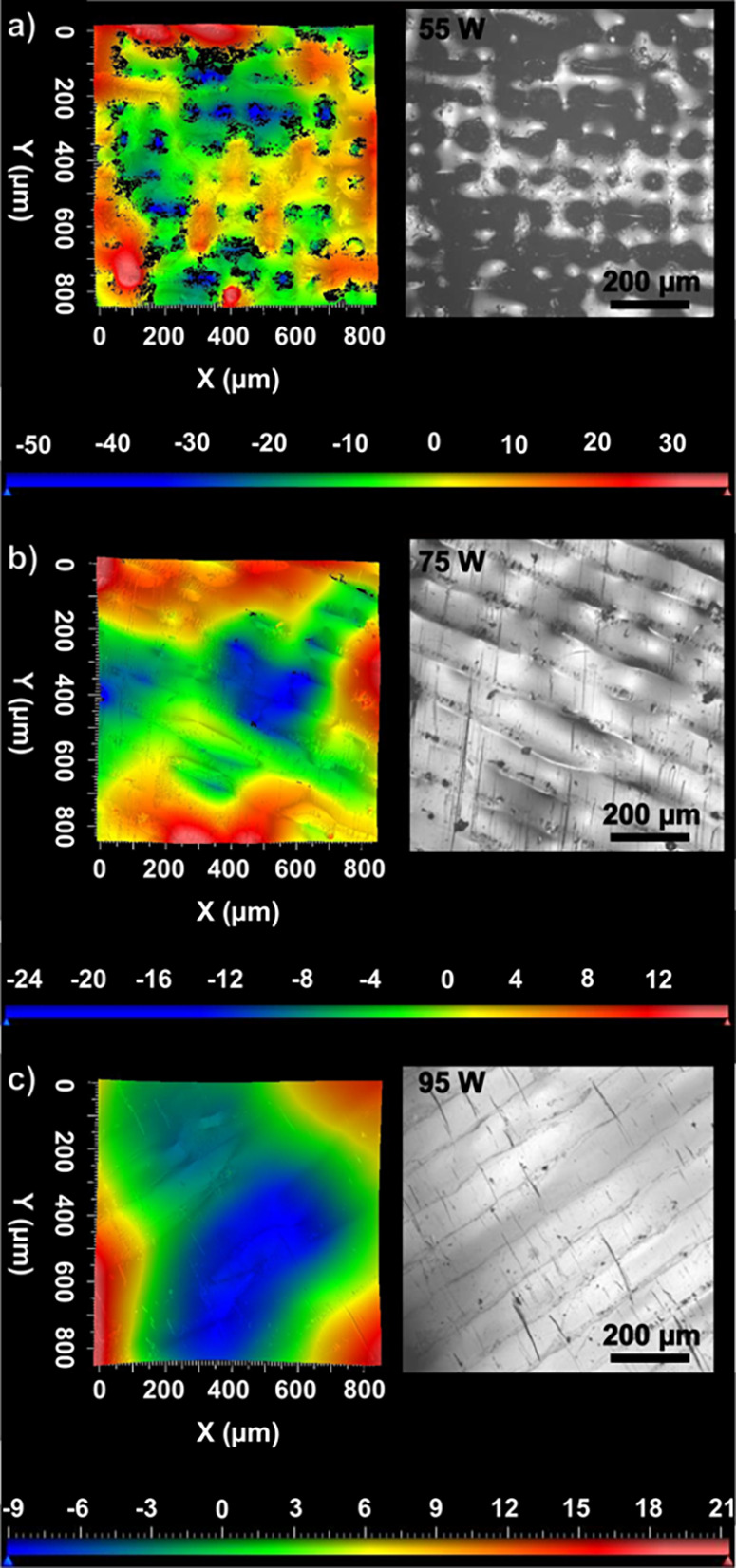
Reconstructed and optical surface images obtained using white light interferometry in as-printed AMLOY-ZR01 samples processed with (a) 55, (b) 75, and (c) 95 W illustrating the change in surface morphology and surface roughness with laser power.

The surface roughness of the as-printed samples was measured using white light interferometry. A decrease in surface roughness with an increase in laser energy density was observed, with the average surface roughness (*S*_a_) changing from 11.6 μm in 55 W to 6.6 μm in 75 W and 4.6 μm in the sample processed using 95 W (Table 1), while the as-cast sample has significantly lower surface roughness, with an *S*_a_ value of 0.12 μm. Figure 2 shows the surface characteristics of as-printed samples with respect to the laser power. The reconstructed image of the 55 W samples exhibits rougher surface with defects such as porosity and partially melted powder tracks. The black features obtained in Figure 2 correspond to regions from which data could not be obtained. The absence in signal arises from surface structures acting as photon traps, absorbing the light. The prevalence of these features is observed to decrease significantly with increasing laser energy density, as shown in Figure 2.

**Table 1:** Surface Roughness of the As-Printed AMLOY-ZR01 Samples and the AMLOY-ZR01 Samples after Completion of the Ion Release Study for a Period of 14 Days (Regular Physiological Condition) and 7 Days (Inflammatory Condition) in Solution^a^

AMLOY-ZR01	as-printed condition		regular physiological conditions		inflammatory conditions	
samples	*S*_a_ (μm)	*S*_z_ (μm)	*S*_a_ (μm)	*S*_z_ (μm)	*S*_a_ (μm)	*S*_z_ (μm)
55 W	11.6 ± 0.4	129.8 ± 10.0	6.7 ± 0.5	82.0 ± 1.0	6.6 ± 0.1	83.0 ± 3.0
75 W	6.6 ± 0.3	68.0 ± 5.0	4.2 ± 0.1	62.0 ± 1.0	4.2 ± 0.1	65.5 ± 0.8
95 W	4.6 ± 0.4	46.0 ± 8.0	2.6 ± 0.4	29.0 ± 3.0	2.3 ± 0.2	28.2 ± 2.0

a S_a_ is the arithmetical mean height and S_z_ the maximum height.

**Figure 3:**
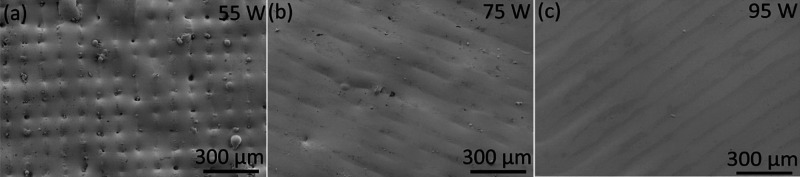
Secondary electron SEM images showing the different top surface morphologies obtained in as-printed AMLOY-ZR01 samples with (a) 55, (b) 75, and (c) 95 W laser power values on selective laser melting. The surface morphology of the as-printed samples and its associated laser tracks are observed to change from a porous to well defined groove-like structure with an increase in laser power.

The surface morphology of the as-printed samples was investigated using SEM, see Figure 3. Porosity and incomplete melting of the material is observed when processing the material at 55 W. However, a significant decrease of unmelted powder particles adhering to the surface of laser printed AMLOY-ZRO1 samples is observed when increasing the laser power (75 W and 95 W). Consequently, this allows for better spreading successive powder bed layers over the surface of the melted layer^49^ and further decreases the amount of unmelted particles present in the sample. Another feature that can be observed from the SEM imaging of as-printed samples is the difference in morphology of the laser tracks formed at different laser powers. Laser tracks formed at 55 W consists of an array of pores arising as a result of lack of fusion and unmelted particles along the track length. Meanwhile, with an increase of laser power (75 and 95 W samples), it is observed that the above-mentioned features associated with lower laser scan power disappear and the tracks attain a smoother groove-like morphology with a well-defined track width as observed from SEM images in Figure 3 and optical interferometry (Figure 2). From the SEM images and optical interferometry measurements (shown in Table 1), it can be emphasized that the major difference in surface morphology and the average surface roughness observed is between the lower power 55 W and the other two high power (75 and 95 W) as-printed samples. The choice of process parameters influences the obtained materials properties, where the surface roughness is assumed to be the most relevant parameter in the current context. The laser power is used as a marker for the samples throughout the publication, with the lowest laser power corresponding to the highest surface roughness. It should be mentioned that other process parameters in addition to the laser power investigated in this work could be further exploited to tailor the surface roughness of the as-printed samples. Hatch spacing between the laser scan vectors, laser scanning strategies, and scan speed are examples of process parameters that may have an effect on the surface roughness of laser-printed AMLOY-ZRO1 samples.^32^

### 3.2 *In Vitro* Cell Studies

In order to investigate the potential of the additively manufactured BMG AMLOY-ZR01 as an orthopedic implant biomaterial, the *in vitro* response of MC3T3-E1 cells to the alloy was studied, together with the impact of the BMG surface roughness and morphology on cell behavior. The first step in the biocompatibility evaluation included the investigation of the potential toxic effects of alloy extracts on the preosteoblastic cells (indirect cytotoxicity test), followed by the evaluation of cell proliferation and differentiation when the BMG served as cell culture substrate.

**Figure 4:**
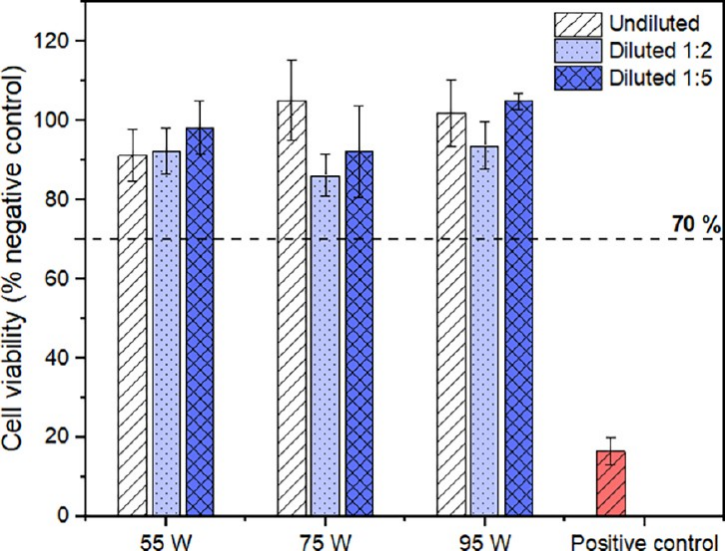
Cell viability of MC3T3-E1 cells cultured with extracts of the AMLOY-ZR01 materials. The data are expressed as percentage of negative control, corresponding to cells cultured in cell culture medium. The positive control corresponds to cells exposed to cell culture medium containing 5% DMSO. Data are presented as mean ± standard error of the mean of four independent experiments. Cell viability greater than 70% indicates no cytotoxic effect.

The results of the indirect cytotoxicity test indicated that the extracts of the as-printed samples did not have a cytotoxic effect, since cell viability values were above the 70% cytotoxicity limit^48^ for all test conditions (Figure 4). The positive control showed a cell viability of <20%, confirming its cytotoxic effect. Furthermore, no statistically significant differences were found between the extracts of the different surface roughness materials. Thus, the outcomes of the indirect cytotoxicity test eliminate concerns about the potential toxic effect of leachables on the cell response in direct contact studies.

**Figure 5:**
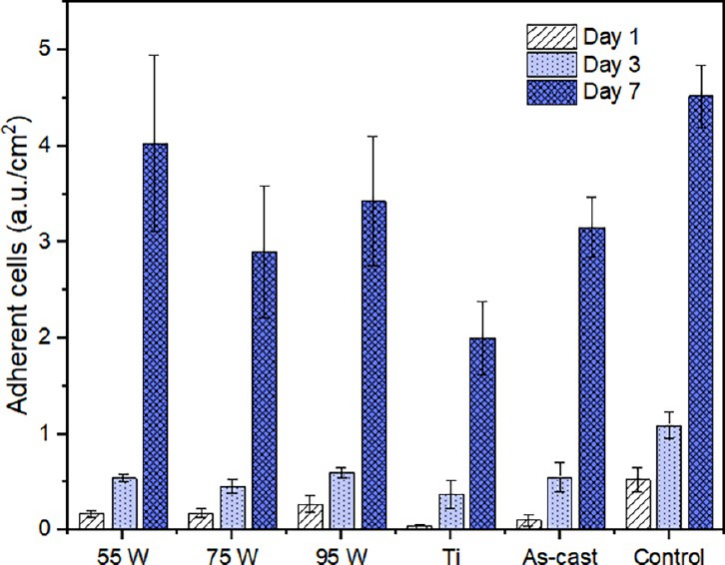
Cell proliferation evaluated by measuring the activity of cytosolic LDH. Results are presented as adherent cells (arbitrary units) normalized over the surface area of the substrate. Control corresponds to cells cultured on TCP (tissue culture plate). Data are presented as mean ± standard error of the mean of at least three independent experiments with triplicate samples.

The response of the MC3T3-E1 cells to the AMLOY-ZR01 materials was evaluated in terms of cell proliferation and differentiation when cells were cultured on the surface of the samples. The results from the proliferation study showed that the cells proliferated over time on the BMG substrates, irrespective of the surface roughness and showed proliferation patterns comparable with the one observed in the control (cells cultured on TCP) (Figure 5). The activity of cytosolic LDH measured across all sample groups demonstrated the same cell proliferation behavior (no statistically significant differences between the groups were observed) irrespective of surface roughness investigated in this study.

**Figure 6:**
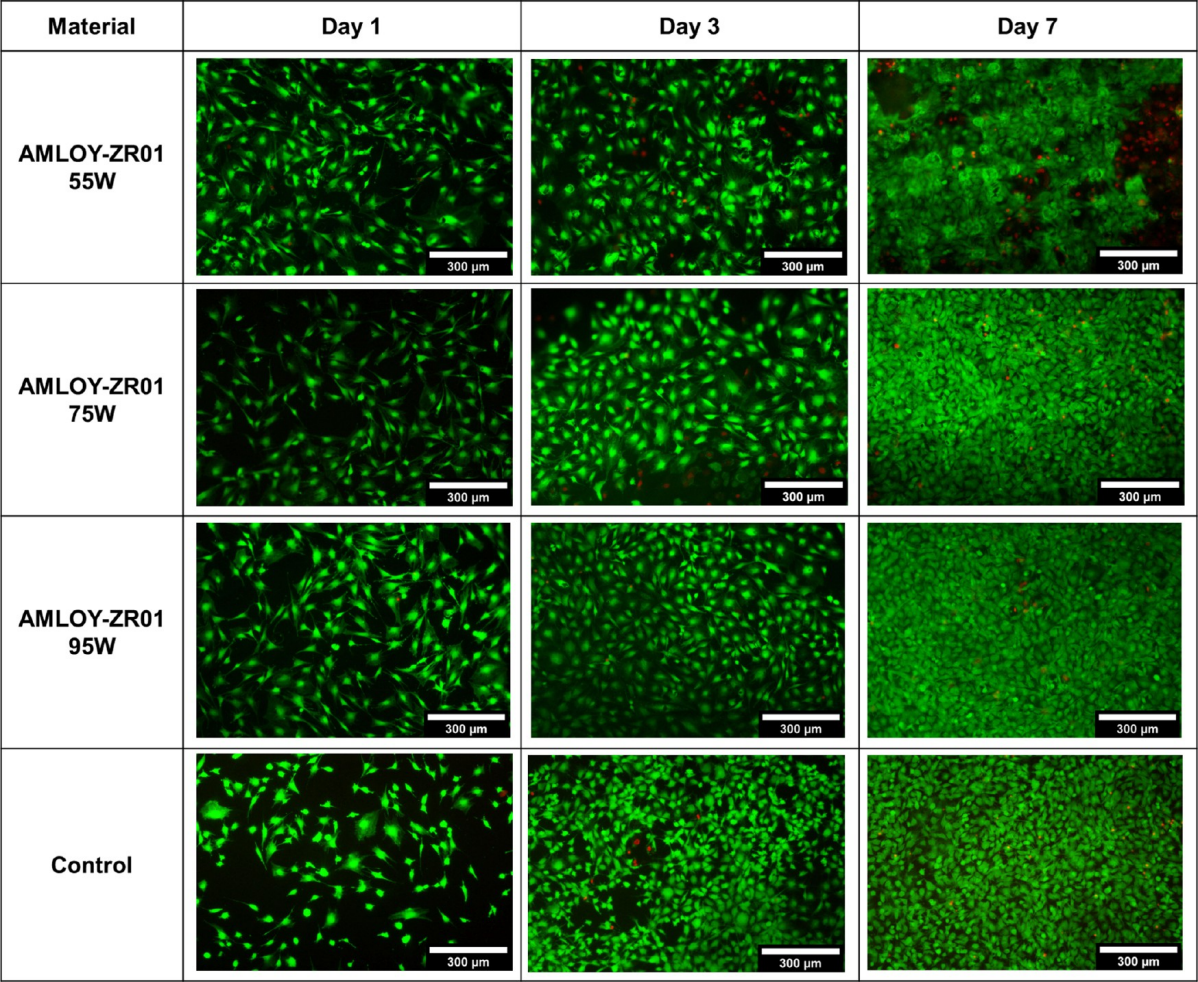
Representative live/dead staining micrographs of cells on the different substrates (55, 75, and 95 W and control) after 1, 3, and 7 days of cell culture. Note that the live/dead staining labels viable cells are green and cells with compromised cell membrane integrity are red.

The analysis of the microscopy images of live/dead stained cells confirmed the proliferation of the cells on the AMLOY-ZR01 substrates. Images showed good cell viability on the different substrates, with a high proportion of viable cells (green cells) compared with those with compromised cell membrane (red cells) for all time points (Figure 6). The exception seemed to be the cells cultured on the 55 W substrate for 7 days, where a relative high number of nonviable cells are revealed beneath a dense layer of viable cells. This distinct pattern is likely a consequence of cells reaching over confluency and easily peeling off from the substrate during the staining process and microscopy imaging.

**Figure 7:**
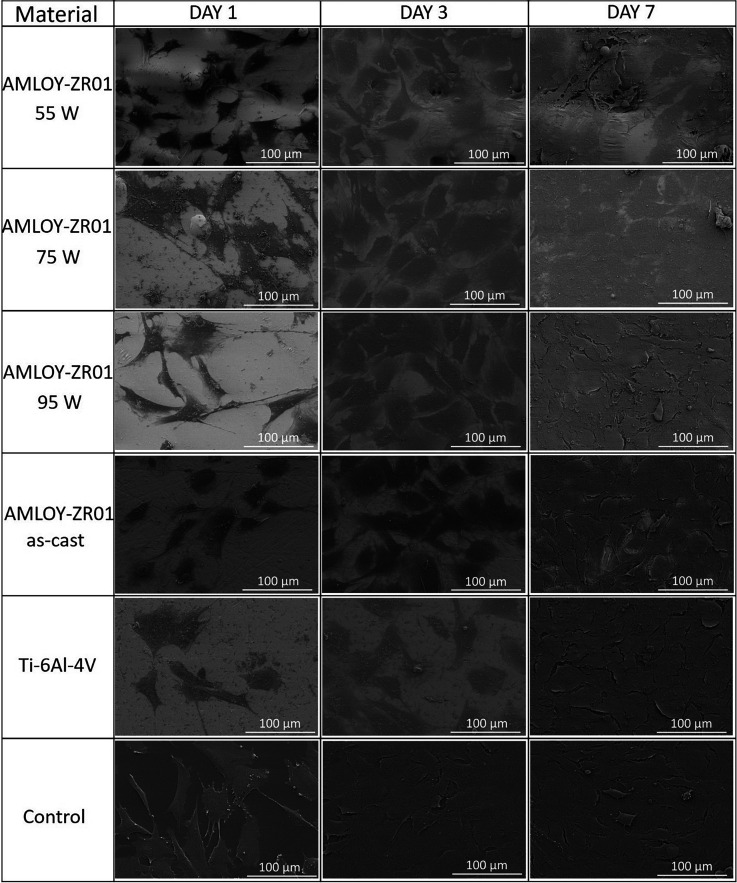
Representative SEM micrographs of cells on the different substrates (55, 75, and 95 W, as-cast, Ti-6Al-4V, and control) after 1, 3, and 7 days of cell culture. The MC3T3-E1 cells are observed to adhere, spread, and proliferate across all the different substrates under the present study.

The SEM imaging of the cells on the different substrates allowed for detailed observation of the cell morphology. Cells showed spread, polygonal shape morphology on all AMLOY-ZR01 substrates as well as on the reference Ti-alloy and on the control (Figure 7). It was observed that cells displayed filopodia and interacted with each other already at day 1, for all the materials under study. After 7 days, a dense cell layer is observed on the surface of the substrates, confirming that the AMLOY-ZR01 samples supported osteoblast growth, irrespectively of the surface roughness. At this time point, cellular boundaries were more difficult to detect in the case of the 55 and 75 W samples compared with the other substrates.

**Figure 8:**
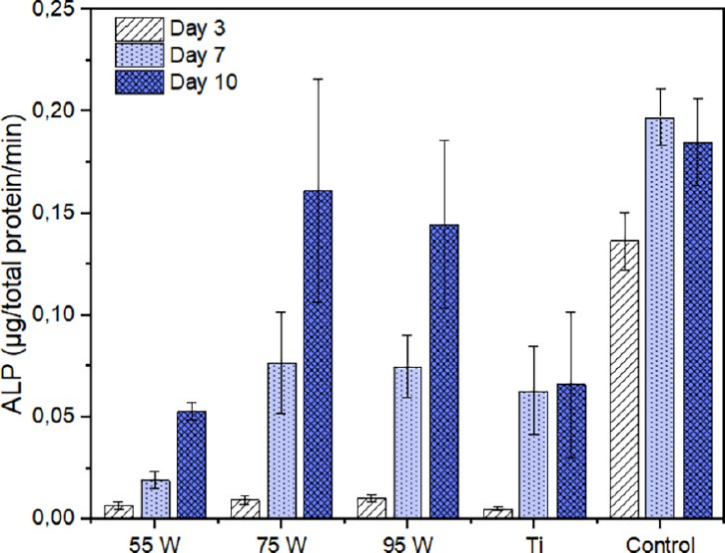
ALP activity of the cells on the different substrates after 3, 7, and 10 days of differentiation. Data are presented as mean ± standard error of the mean of at least three independent experiments with triplicate samples.

To evaluate the differentiation capacity of the MC3T3-E1 cells when cultured on the AMLOY-ZR01 materials, cells were allowed to proliferate on the surface of the substrates during 3 days and thereafter induced to differentiate into osteoblasts. The extent of cell differentiation was evaluated by measuring the ALP activity of the adherent cells 3, 7, and 10 days after the addition of the differentiation factors. Results indicated that ALP activity increased with time for the AMLOY-ZR01 substrates, with higher ALP activity observed with the cells cultured on the 75 and 95 W substrates compared with the 55 W discs after 7 and 10 days of differentiation (Figure 8). However, the significance of such differences was not confirmed by the statistical analysis.

Overall, the biocompatibility evaluation indicated that the AMLOY-ZR01 substrates supported cell proliferation and differentiation of the preosteoblastic cells MC3T3-E1, while no distinct cell response was observed when comparing the different surface roughnesses. Moreover, the cell behavior on the BMG alloy was comparable to the results obtained with the reference material Ti-6A1-4V. The promising cell response to AMLOY-ZR01 is in accordance with previous biocompatibility studies on Zr-based BMGs,^50−52^ demonstrating that the additively manufactured AMLOY-ZR01 alloy is a good contender among other BMGs. Regarding the effect of surface roughness on cell response, a few studies have investigated how the surface roughness of Zr-BMGs may modulate the behavior of bone forming cells.^43,45,53^ Studies by Huang et al. and Li et al. found that surfaces with roughness in the microscale (Ra values in the order of 0.2 to 2.1 μm) on Zr-BMGs favored proliferation and differentiation of bone cells in comparison with roughness in the nanoscale (Ra values in the order of 14 and 73 nm).^43,53^ However, Blanquer et al. investigated different surface topographies with different degrees of surface roughness (from nanoroughness to macroroughness) and concluded that surface roughness and morphology played a minor role on cell–material interactions,^45^ results that are in line with our findings. Differences not only in the level of surface roughness (microroughness vs nanoroughness) but also differences in surface chemistry and surface morphology between the materials under investigation may explain the contradictory findings among the different studies. This highlights the challenges in determining correlations between material surface roughness and the response of bone cells, and points out the need of case-by-case studies. For the additively manufactured AMLOY-ZR01, tailoring surface microroughness and surface morphology (porous or groove-type laser tracks) did not have a significant effect on modulating the response of the MC3T3-E1 cells.

### 3.3 Ion Release Study

The physiological environment and the specific host response to a metallic implant may influence the degradation of the metallic biomaterial overtime.^35^ For this reason, the ion release from the as-printed AMLOY-ZR01 samples was characterized under two separate environments; viz. regular physiological conditions and simulated inflammatory conditions. When simulating regular physiological conditions, it is important to include proteins in the medium, since under an *in vivo* situation, the implanted biomaterial will be covered by a layer of proteins and the presence of such a layer may influence the metal ion release from the implant.^34^ Moreover, it is relevant to consider the effect of the harsh environment that the biomaterials will be exposed to during the first days after implantation. Inflammatory reactions are prone to occur in the tissue surrounding the implant upon implantation, with local inflammation leading to altered conditions (i.e., drastic drop in the pH level close to the implant and high levels of ROS such as peroxides, hydroxyl radicals, and superoxides).^54^ The biocorrosion of various BMGs have been investigated, with the amorphous alloys generally demonstrating a higher corrosion resistance than their crystalline counterparts in simulated body fluids.^28,55−57^ While several studies have evaluated the corrosion behavior of various metals and metal alloys in simulated inflammatory conditions,^58,59^ to the authors’ knowledge, this is the first time the corrosion behavior of BMGs in biofluids containing proteins and simulated inflammatory conditions is investigated.

The corrosion resistance of the samples was studied in the two different electrolytes, i.e., simulated physiological and inflammatory conditions, under open circuit potential conditions (OCP) for 14 and 7 days, respectively. Under OCP conditions, no external potential is applied to the studied material. The OCP is referred to a mixed potential involving anodic and cathodic reactions taking place on a passive layer that was already present on the sample surface prior to the experiment.

**Figure 9:**
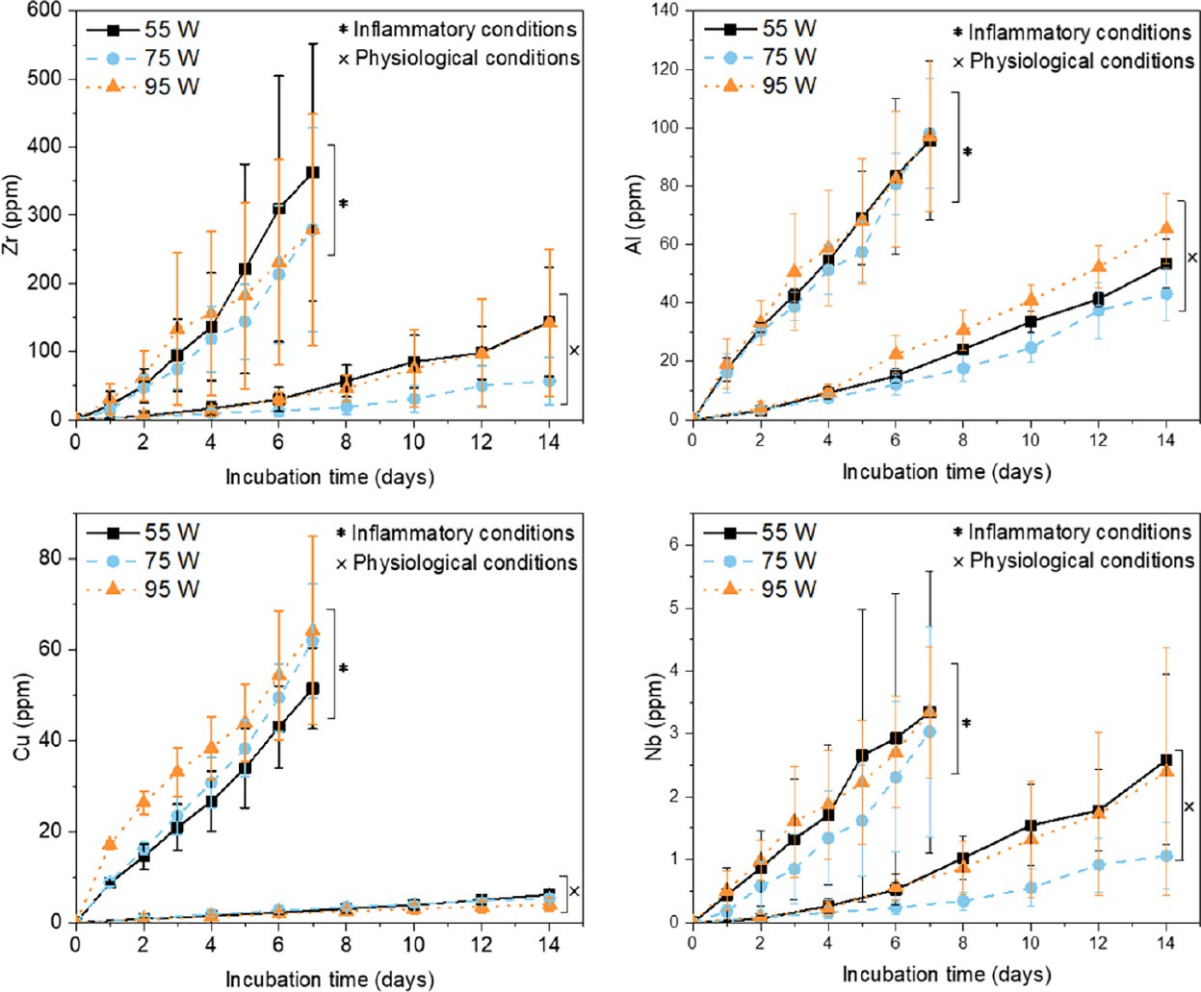
Accumulative ion release from the as-printed AMLOY-ZR01 materials (55, 95, and 74 W) under regular physiological conditions and simulated inflammatory conditions. Data represent three independent experiments for each condition.

After 14 days of immersion tests under OCP in simulated physiological conditions, the results from the ion release study showed different amounts of ions in the electrolyte solution for each metallic element (Figure 9). The highest ion concentration was found for Zr (around 50–150 ppm), following by that of Al (around 30–60 ppm), and a lower ion concentration was found for Cu (around 5 ppm) and Nb (between 1 and 2 ppm) (Figure 9). Considering the lower standard potentials of Zr (i.e., *E*^0^ = −1.45 V vs SHE) and Al (i.e., *E*^0^ = −1.66 V vs SHE) compared to that of Cu (i.e., *E*^0^ = 0.34 V vs SHE) and Nb (i.e., *E*^0^ = −1.09 V vs SHE), the leaching of Zr and Al happens more readily than the other two metals (standard potential values for the reduction reactions of the relevant oxides are also given in the Supporting Information). To support the ion release studies, complementary high-resolution XPS spectra for the Nb 3d, Zr 3d, Cu 2p, and Al 2p core-level regions were obtained on a 55 W as-printed sample, which is the sample with the higher surface roughness, prior and after the incubation periods. The XPS measurements are displayed in Figure S1 in the Supporting Information (SI). The spectra were recorded without any presputtering process and showed that the surface oxide layer of the as-printed sample (blue curves) was mostly Zr-rich and free from Nb. The Al 2p and Cu 2p signals were less intense indicating probably the presence of Al and Cu as traces in the native oxide. The observed Zr-rich native oxide layer prior to the OCP experiment agrees with the high observed Zr ion release from the sample surface. The fact that Zr and Al have a lower standard potential with respect to Cu and Nb indicates that the etching process can be facilitated for these two elements. Etching of Cu was observed but in a lower concentration in the electrolyte than Zr or Al (Figure 9). The surface of the as-printed sample was not rich in Cu, and therefore, this observation goes along with the low ion concentration in the electrolyte after the incubation period. Considering that the native oxide was free from Nb and the fact that Nb is considered as a main passive element of the composition (i.e., according to the Pourbaix diagrams^60^), the Nb ion concentration was barely seen in the electrolyte (Figure 9). The XPS spectra after 14 days of incubation under simulated physiological conditions (Figure S1, green curve) showed that the oxide components include Al, Zr, and Cu but not Nb. The fact that Nb was not detected by XPS implies that this element is absent from the oxide layer and justifies the limited amount of Nb seen in the ion metal release study.

A similar OCP experiment was also performed on the 55, 75, and 97 W samples after 7 days of incubation in simulated inflammation conditions. The main difference between the simulated physiological and the inflammatory conditions concerns the addition of H_2_O_2_ in the electrolyte along with the addition of Cl^–^ ions. A higher ion release was observed after the incubation period under the inflammatory conditions compared to physiological conditions (Figure 9). Although, the ion release trend was maintained as Zr > Al > Cu > Nb similarly to physiological conditions (Figure 9), the ion release results under inflammatory conditions showed that the most significant ion increase corresponded to Cu, with over 10 times higher ion concentration (i.e., about 60 ppm), compared to regular physiological conditions. This observation agrees with the Cu Pourbaix diagram,^60^ where copper can be dissolved in Cu^++^ species and transferred to the electrolyte media rather than forming a stable passive layer under low pH conditions (i.e., pH 5.2 in inflammatory conditions). The XPS high-resolution spectra on the 55 W sample surface after 7 days of incubation under simulated inflammation conditions (Figure S1, red line), showed no signal for Cu, but complementary SEM-EDS mapping for the same sample (see Figure S2 in the SI) showed some local copper segregation on the sample surface. Regarding the rest of the metals, a homogeneous elemental distribution down to the μm-scale according to SEM/EDS maps was seen, without observing any elemental segregation around the pores by using this technique.

The local corrosion attack under inflammatory conditions was directly reflected on the increased ion concentration release from the as-printed AMLOY-ZR01 substrates as shown in Figure 9. Comparing the ion release rates found for different AMLOY-ZR01 materials, no clear trend between the degree of surface roughness and ion release rate was found (Figure 9). It was expected that the pores on the surface of the 55 W sample could act as sites for preferred local corrosion and could hence lead to more leaching out of elements from this sample compared with the 75 and 95 W; however, this was not observed in the ion release studies. The fact that the effect of roughness was not significant on the corrosion resistance of the samples under OCP conditions enhance the idea that the corrosion rate was probably controlled by the cathodic reactions.

These findings draw attention to the important question of how the metal ions released from the AMLOY-ZR01 alloy will affect the surrounding tissue under different physiological conditions. As mentioned above, the highest ion release was found for Zr, which is considered as a biocompatible element,^43^ and research studies have found that Zr-ions appear to be able to induce both proliferation and differentiation of primary human osteoblasts.^61^ Regarding the release of Cu and Al, these elements are naturally occurring trace elements in the human body;^62^ however, they could represent a health threat if present at high concentrations.^63^ Nevertheless, the *in vitro* cell studies indicated that the levels of ion release under regular physiological conditions did not have a negative effect on the viability and proliferation of the MC3T3-E1 cells when cultured in direct contact with the materials (Figure 6) or in the indirect cytotoxicity test (Figure 4). Still, future studies should investigate the response of the osteoblastic cells when the cell–material interactions take place in an environment where high levels of ions are expected to be released from the alloy, i.e., an inflammation scenario.

**Figure 10:**
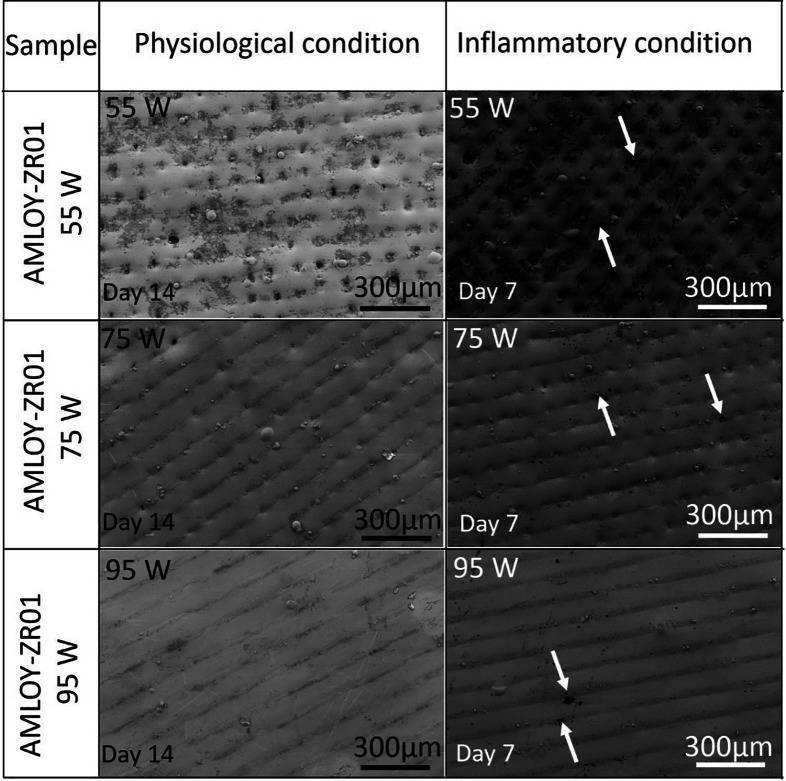
SEM images showing the surface morphology of as-printed samples after incubation under regular physiological and inflammation conditions for 14 and 7 days, respectively. An increase in surface defects after the material exposure to inflammatory conditions is indicated by the white arrows.

**Figure 11:**
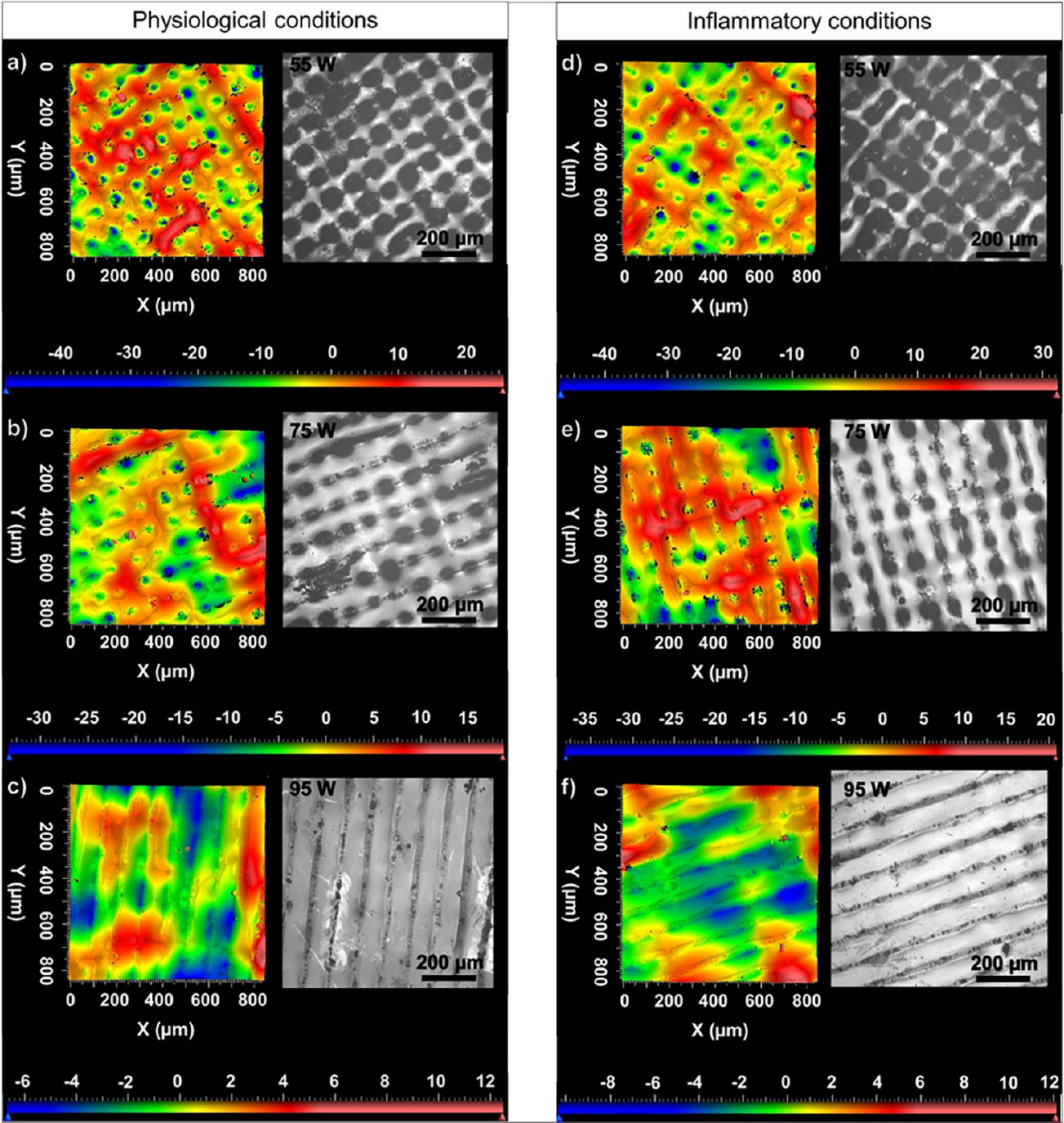
Reconstructed and optical surface images obtained using white light interferometry in as-printed AMLOY-ZR01 samples post ion release studies in regular physiological test conditions (a) 55, (b) 75, and (c) 95 W and inflammatory test conditions (d) 55, (e) 75, and (f) 95 W.

The surface roughness and morphology were evaluated after the ion release study to investigate the effect of material exposure on the simulated conditions on the surface characteristics. A decrease in surface roughness was observed under both conditions, and the average surface roughness (*S*_a_) dropped to nearly half the value, from 11.6 to 6.7–6.6 μm in the as-printed 55 W sample post ion release study, while a less substantial drop of approximately 2 μm in average surface roughness (*S*_a_) was observed in the 75 and 95 W samples (Table 1). An influencing factor leading to the marked drop in the surface roughness of the 55 W sample could possibly be attributed to the etching away of rough surface areas and the subsequent formation of smoother surfaces in the presence of cell culture medium under both regular physiological and inflammatory test conditions. From the reconstructed optical interferometry images shown in Figure 11, together with the SEM images of the surface presented in Figure 10, it can be observed that the 55 W sample exhibits a comparatively smoother morphology after the ion release studies with respect to its corresponding as-printed surface (Figure 2a). The decrease in surface roughness observed after the ion release study in the as-printed 55 W sample could be due to the etching away of surface structures, which acts as photon traps under the influence of the electrolytes as compared to its initial condition illustrated in Figure 2a. Furthermore, the surface morphology of all samples significantly changed after exposure to inflammatory conditions, with an observed increase in local corrosion due to the highly oxidative environment as explained above.

## 4 Conclusions

The present work showed that surface roughness and morphology of the additively manufactured Zr_59.3_Cu_28.8_Al_10.4_Nb_1.5_ BMG can be tailored by tuning the laser power applied in the SLM process. The as-printed BMG exhibited comparable biocompatibility to the orthopedic biomaterial Ti grade 5 alloy, in terms of cell proliferation and differentiation of the preosteoblastic cells MC3T3-E1. The range of surface roughness under study did not have a significant effect on modulating the cell response. The ion release study highlighted the significant effect of inflammatory conditions on increasing the local corrosion of the BMG, irrespective of the surface roughness of the samples. The findings of this study collectively suggest that Zr_59.3_Cu_28.8_Al_10.4_Nb_1.5_ is a potential candidate for fabricating novel implants, including patient-specific such. However, further studies are necessary to elucidate the biological response to Zr_59.3_Cu_28.8_Al_10.4_Nb_1.5_ under inflammatory conditions including elevated ion release from the alloy surface.

## Supporting Information Available

Standard potential values of the metals present in *AMLOY-ZR01* and standard potential values for the reduction reactions of the relevant oxides, X-ray photoelectron spectroscopy analysis of samples before and after the ion release study, and energy-dispersive X-ray spectroscopy mapping of the 55 W sample after exposure to inflammatory conditions (PDF)

## Notes

^#^ Shared last authorship and corresponding authors.

## Notes

^⊥^ Shared first authorship.
