# Supplementary material for: Biocompatibility of a Zr-Based Metallic Glass Enabled by Additive Manufacturing
Source: ACS Appl Bio Mater. 2022 Dec 2;5(12):5741–53. doi: 10.1021/acsabm.2c00764 (PMC9768811; doi:10.1021/acsabm.2c00764)
Supplement: Supplementary file 1 — mt2c00764_si_001.pdf [file mt2c00764_si_001.pdf]

Supporting Information: Biocompatibility of a Zr-based metallic glass enabled by additive manufacturing

Lisa Larsson[1,], Jithin James Marattukalam[2,*], Eirini-Maria Paschalidou[3], Björgvin Hjörvarsson[1], Natalia Ferraz[4, §], Cecilia Persson[1, §].*

[1] Department of Materials Science and Engineering, Biomedical Engineering, Box 534, SE- 75121, Uppsala, Sweden.

[2] Department of Physics, Materials Physics, Uppsala University, Box 530, SE-75121, Uppsala, Sweden.

[3] Department of Chemistry Ångström, Uppsala University, Box 538, SE-751 21 Uppsala, Sweden.

[4] Department of Materials Science and Engineering, Nanotechnology and Functional Materials, Box 35, SE- 75103, Uppsala, Sweden.

[*]Shared first authorship

[§]Shared last authorship and corresponding authors.

Standard potentials

The standard potentials for the existing metals in the studied composition are as follows. The potentials are given vs. standard hydrogen electrode (SHE):

$Al^{3+} + 3\ e^- = Al$ $E^0 = -1.66$ V vs. SHE

$Zr^{4+} + 4\ e^- = Zr$ $E^0 = -1.45$ V vs. SHE

$Nb^{3+} + 3\ e^- = Nb$ $E^0 = -1.09$ V vs. SHE

$Cu^{2+} + 2\ e^- = Cu$ $E^0 = 0.34$ V vs. SHE

The standard potentials for the reactions listed below were estimated using the equation $\Delta G^0 = -nFE^0$ and the following ΔG^0 values; Al_2O_3 (-1582 kJ/mol), ZrO_2 (-1043 kJ/mol), CuO (-130kJ/mol), H_2O (-237 kJ/mol) from *SI Chemical data, 5th edition (2002), G. Aylward & T. Findlay, Wiley* and Nb_2O_5 (-1766 kJ/mol), from *A. Srinath et al. Corr. Science 188 (2021) 109557*. The potentials are given vs. SHE.

$Al_2O_3 + 6\ e^- + 6\ H^+ \rightarrow 2\ Al + 3\ H_2O$ $E^0 = -1.50$ V vs. SHE

$ZrO_2 + 4\ e^- + 4\ H^+ \rightarrow Zr + 2\ H_2O$ $E^0 = -1.47$ V vs. SHE

$Nb_2O_5 + 10\ e^- + 10\ H^+ \rightarrow 2\ Nb + 5\ H_2O$ $E^0 = -0.64$ V vs. SHE

$CuO + 2\ e^- + 2\ H^+ \rightarrow 2\ Cu + H_2O$ $E^0 = 0.55$ V vs. SHE

X-ray Photoelectron spectroscopy

X-ray Photoelectron spectroscopy (XPS) investigations were performed with a PHI Quantera II scanning XPS microprobe using monochromatic Al Kα radiation (*hν* = 1486.7 eV). A spot size of 200 µm and a photoelectron take-off angle of 45° were used in all measurements.

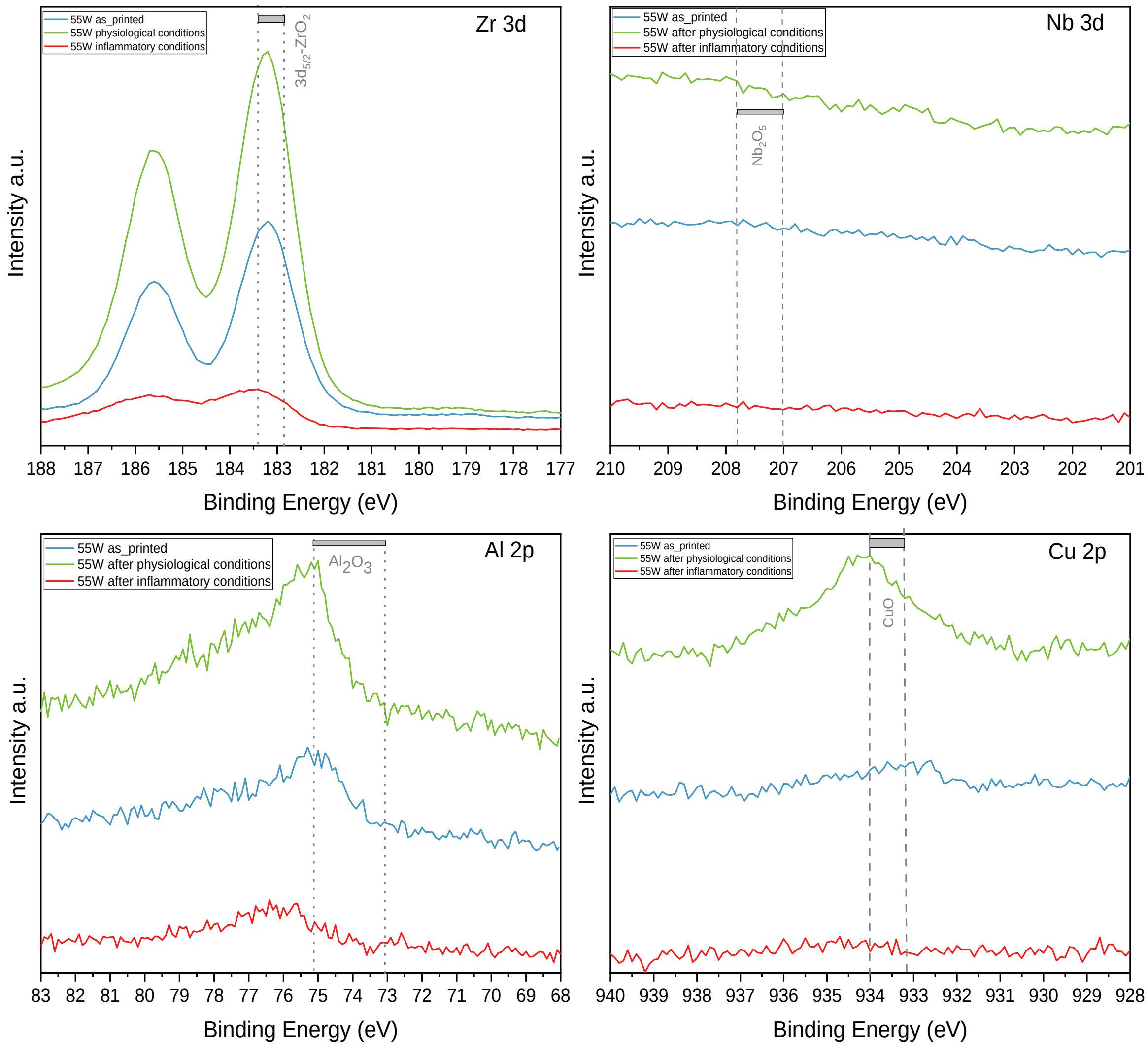

Figure S1: High-resolution XPS spectra for the Nb 3d, Zr 3d, Cu 2p and Al 2p were obtained for the 55W as-printed (blue curves), after physiological (green curves) and inflammatory (red curves) conditions for 14 and 7 days respectively days. The binding energy range for the oxides marked in the spectrum are based on reference: *Handbook of X-ray photoelectron spectroscopy, Perkin-Elmer Corporation-Physical Electronics division (1992).*

Energy-dispersive X-ray spectroscopy

A Zeiss 1530 SEM equipped with an EDS detector was used at a working distance of 8.5 mm with an electron high current of 10 keV was used in the (EDX) analysis. The Oxford AZtec (INCA energy) software was used for elemental mapping.

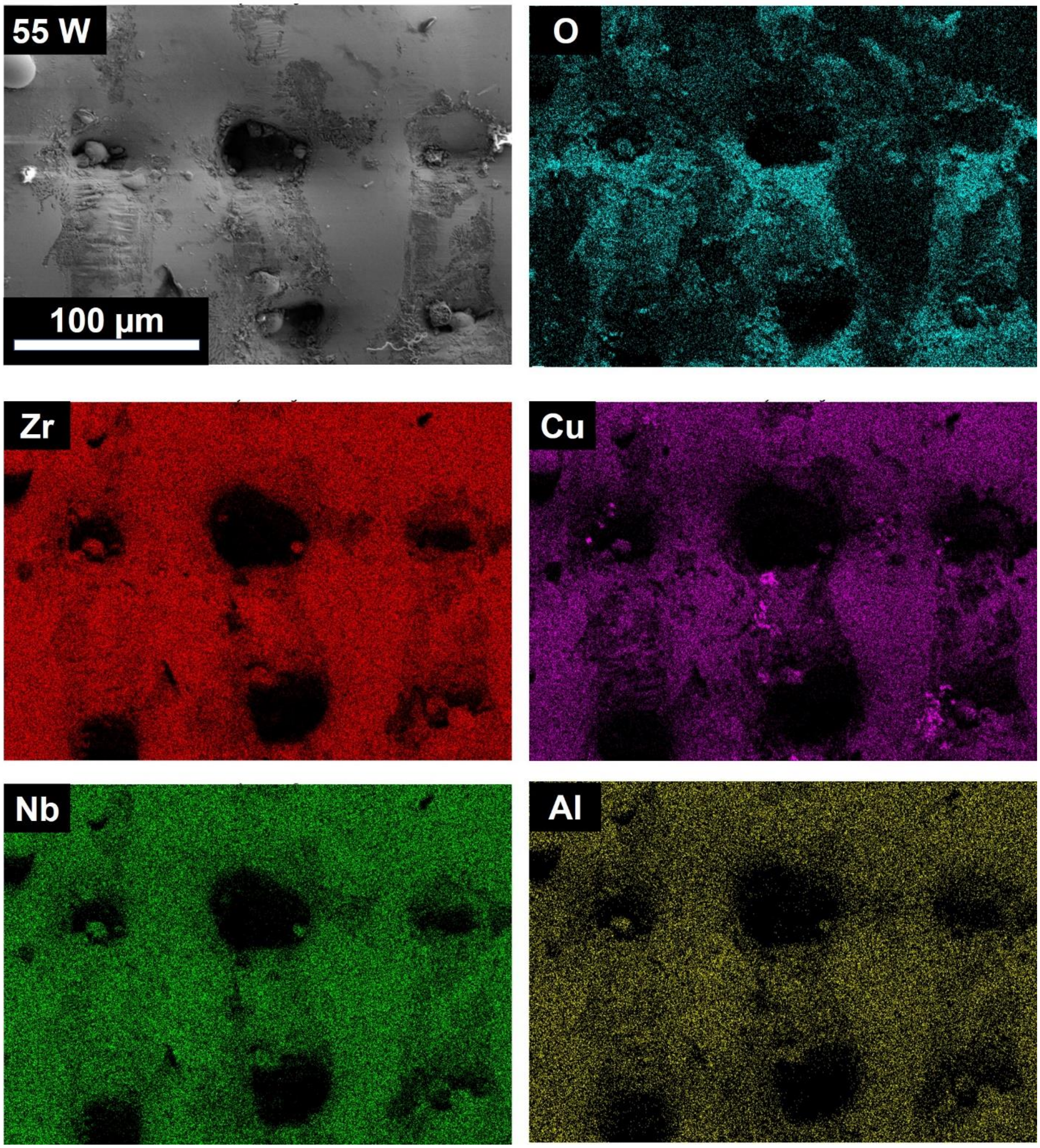

Figure S2: Results of EDX mapping of 55W sample after immersion in inflammatory conditions for 7 days.
